# Clinical factors within a week of birth influencing sodium level difference between an arterial blood gas analyzer and an autoanalyzer in VLBWIs: A retrospective study

**DOI:** 10.1097/MD.0000000000028124

**Published:** 2021-12-10

**Authors:** Hyun Ho Kim, Jin Kyu Kim

**Affiliations:** a Department of Pediatrics, Jeonbuk National University School of Medicine, Jeonju, South Korea; b Research Institute of Clinical Medicine of Jeonbuk National University-Biomedical Research Institute of Jeonbuk National University Hospital, Jeonju, South Korea.

**Keywords:** blood gas analysis, hyponatremia, physiologic weight loss, preterm infant, sodium

## Abstract

Neonatologists often experience sodium ion level difference between an arterial blood gas analyzer (direct method) and an autoanalyzer (indirect method) in critically ill neonates. We hypothesize that clinical factors besides albumin and protein in the blood that cause laboratory errors might be associated with sodium ion level difference between the 2 methods in very-low-birth-weight infants during early life after birth. Among very-low-birth-weight infants who were admitted to Jeonbuk National Hospital Neonatal Intensive Care Units from October 2013 to December 2016, 106 neonates were included in this study. Arterial blood sample was collected within an hour after birth. Blood gas analyzer and biochemistry autoanalyzer were performed simultaneously. Seventy-six (71.7%) were found to have sodium ion difference exceeding 4 mmol/L between 2 methods. The mean difference of sodium ion level was 5.9 ± 6.1 mmol/L, exceeding 4 mmol/L. Based on sodium ion level difference, patients were divided into >4 and ≤4 mmol/L groups. The sodium level difference >4 mmol/L group showed significantly (*P* < .05) higher sodium level by biochemistry autoanalyzer, lower albumin, lower protein, and higher maximum percent of physiological weight than the sodium level difference ≤4 mmol/L group. After adjusting for factors showing significant difference between the 2 groups, protein at birth (odds ratio: 0.835, 95% confidence interval: 0.760–0.918, *P* < .001) and percent of maximum weight loss (odds ratio: 1.137, 95% confidence interval: 1.021–1.265, *P* = .019) were factor showing significant associations with sodium level difference >4 mmol/L between 2 methods. Thus, difference in sodium level between blood gas analyzer and biochemistry autoanalyzer in early stages of life could reflect maximum physiology weight loss. Based on this study, if the study to predict the body's composition of extracellular and intracellular fluid is proceeded, it will help neonatologist make clinical decisions at early life of preterm infants.

## 1 Introduction

Measuring sodium ion level in blood is essential for preterm infants because sodium imbalance can lead to convulsions and brain damage.^[1–3]^ Therefore, accurate sodium measurement is one of the most important requirements in neonatal intensive care unit (NICU). However, neonatologists often experience different sodium ion levels between direct and indirect ion-selective electrode (ISE) methods for preterm infants. In a previous study, we have found that such difference in sodium ion level is associated with serum protein level like adult patients.^[4]^ Most of previous studies have also shown that sodium ion difference can be caused by hypoproteinemia and/or hypoalbuminemia.^[5–7]^ And sodium ion difference is often associated with critically ill conditions requiring ICU admission.^[8,9]^

Preterm infants in the early stage of life have physiologic weight loss different from children and/or adults. They may also have unstable vital sign, immature kidney, neonatal morbidities, and death. Moreover, sodium ion level difference exceeding 4 mmol/L between direct and indirect ISE method is often seen in ICU, especially in very-low-birth-weight infants (VLBWIs) during early life after birth.^[4,7]^ The United States Clinical Laboratory Improvement Amendments standard for the difference between electrolyte tests recommends that sodium ion level difference between any 2 test methods should not exceed 4 mmol/L.^[10]^ Despite efforts to achieve accurate examination by eliminating difference of sodium ion level, discrepancy remains unsolved, making it difficult for neonatologists to diagnose and treat sodium imbalance, especially in preterm infants during early life after birth.

In the current study, in addition to known causes of protein and albumin in blood, clinical factors within the first week of life relevant to sodium ion differences between a blood gas analyzer using direct ISE method and biochemistry autoanalyzer using indirect ISE method in VLBWIs were determined. We hypothesize that clinical factors or characteristics of VLBWIs during early life after birth are associated with sodium ion difference between direct and indirect ISE methods.

## 2 Subjects and methods

The present study included inborn preterm infants who were admitted to Jeonbuk National Hospital NICU from October 2013 to December 2016. Data of VLBWIs (birth weight <1500 g) who underwent arterial blood collection for electrolyte analysis were retrospectively reviewed. Blood test data were collected through electronic medical record chart review. Blood tests were conducted on the first day and the first week. Clinical features of VLBWIs within the first week of life were obtained. First, patients who had hemolytic samples on the first day were excluded from this study. Second, we also excluded patients who used diuretics or steroids that could affect urine amount within a week of life. Third, infants diagnosed as congenital anomalies were excluded. The Jeonbuk National Hospital Institutional Review Board approved this study. The requirement of informed consent was waived due to the retrospective nature of this study.

Arterial blood sample was collected within an hour after birth in NICU. Blood tests including blood gas analyzer as a direct ISE method and biochemistry autoanalyzer as an indirect ISE method were performed using the simultaneously collected arterial blood. All samples (1–2 mL arterial blood samples) of patients were divided into a dry heparin syringe (BD PresetTM, BD Diagnostics, Plymouth, UK) and a microtube (MicrotainerTM tubes #365978, BD Diagnostics) and sent to the Division of Laboratory Medicine (central laboratory assay). The sample in the dry heparin syringe was analyzed with a benchtop blood gas analyzer (Stat Profile CCX Series, Nova Biomedical, Waltham, MA) using the direct ISE method. The blood gas analyzer undergoes an automatic 2-point calibration every 2, 4, or 6 hours and a single-point calibration every 30 minutes or after each sample is input. Another sample placed into the microtube was analyzed with a biochemistry auto-analyzer (ADVIA 2400 Clinical Chemistry System, Siemens, Tarrytown, NY) using the indirect ISE method. This machine contains an auto-calibration system that maintains the accuracy and precision using a sample buffer. Each sample was tested within 10 minutes after being put in the test container.

All preterm infants in NICU were managed with the following fluid management protocol. Fluid replacement for patients was commenced at 60 mL/kg for the first day of birth and gradually increased to 120 mL/kg until 10 days after birth. Daily fluid requirements were calculated considering urine output and insensible water loss (IWL). Considering the calculated daily requirement, the input volume was increased within the range of 10 to 15 mL/kg per day. From the first day of birth, tropic feeding (<20 mL/kg) was started. Feeding volume was increased based on feed tolerance. If the amount of feeding did not increase, then parental nutrition was supplemented. All processes of fluid and nutrition management were decided by attending neonatologists. During a week after birth, all VLBWIs were managed in incubators (Giraffe OmniBed, General Electric, Boston, MA). To control additional IWL from the skin and mucus membrane, babies were wrapped in plastic wraps right after birth. A high humidity was provided to the incubator. Each patient's weight was measured in the incubator at 6 am by trained nurses. To record the weight exactly, the tube and line were held by nurses and the sensor attached to the patient was removed before the weight of infant was measured. If the patient's weight differed by more than 10% compared to the previous day, it was measured again after zeroing the scale. Humidity and temperature of the incubator were adjusted according to neutral thermal environments. They were adjusted at the discretion of the neonatologist.^[11]^ A patient with less than 25 weeks of gestational age (GA) was cared for with minimal handling to reduce IWL due to immature skin. Phototherapy was applied according to the result of daily total bilirubin.

VLBWIs were divided into 2 groups based on sodium ion level difference of 4 mmol/L (the United States Clinical Laboratory Improvement Amendments recommendation) between blood gas analyzer as a direct ISE method and biochemistry autoanalyzer as an indirect ISE method on the first day after birth.^[10]^ Results of blood tests on the first day and the seventh day included electrolyte, protein, and albumin. Perinatal demographics and maternal factors and preterm infants’ clinical factors within the first week of life were collected. Perinatal demographics characteristics included GA, birth weight, sex, 1-minute Apgar score, 5-minute Apgar score, and small for gestational age (SGA). Maternal factors included maternal age, multiple gestations, completed antenatal steroid, IVF (in vitro fertilization), maternal antibiotics, clinical chorioamnionitis, gestational diabetes mellitus, pregnancy-associated hypertension, and preterm premature rupture of membrane. Vital signs, chemistry, and electrolyte on the first day of birth were collected. Neonatal mortality predicting scores at birth, including clinical risk index of babies II score, SNAP (score for neonatal acute physiology) II score, and SNAP perinatal extension score, were also collected. Preterm infants’ diseases within the first week of life included high grade intraventricular hemorrhage (IVH) (grade 3-4 IVH), respiratory distress syndrome (RDS), hemodynamically significant patent ductus arteriosus (PDA), blood culture proven early sepsis, maximum percentage of weight loss, input volume, amount of urine output, duration of ventilator care, duration of phototherapy, and death within 7 days.

### 2.1 Statistical analysis

Two groups categorized by sodium ion level difference of 4 mmol/L of between direct and indirect ISE method were compared with unpaired *t*-test. Deming regression analysis and a Bland–Altman plot were used to evaluate sodium ion level differences between the 2 ISE methods. To find clinical factors associated with sodium ion level difference between the 2 ISE methods, logistic regression analysis was conducted. Univariate logistic regression analysis was performed for clinical factors and blood tests within a week of life. We determined whether clinical factors and blood test variables that showed a difference in univariate analysis at *P* < .1 might be associated sodium ion level differences by multivariate regression analysis. All analyses were performed using SPSS version 24.0 (IBM Corp., Armonk, NY) and MedCalc Statistical Software version 16.0 (MedCalc Software, Mariakerke, Belgium). A *P-*value of less than .05 was deemed statistically significant.

## 3 Results

**Figure 1:**
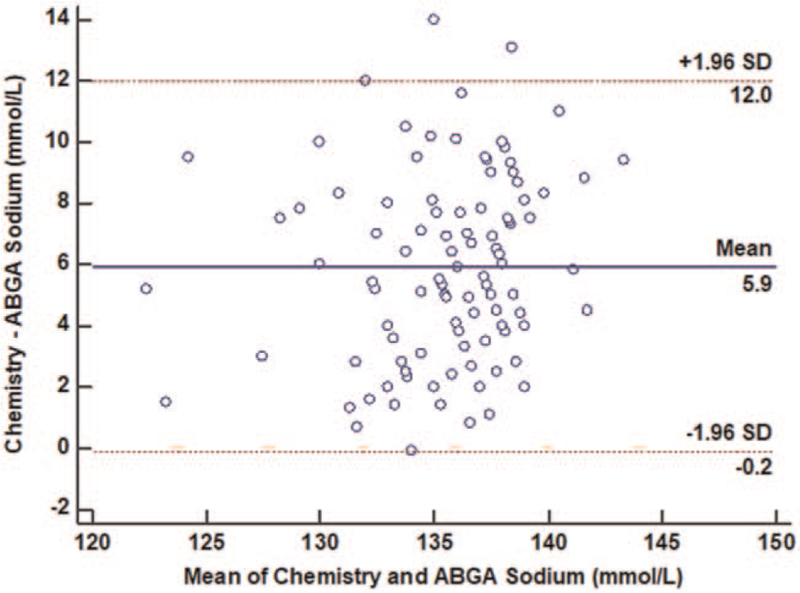
Bland–Altman plot graph using chemistry and ABGA sodium ion level. Mean of chemistry and ABGA sodium ion are presented on the X-axis. The mean sodium ion level difference between chemistry and ABGA was 5.9 ± 6.1 mmol/L. ABGA = arterial blood gas analysis.

Sodium ion levels of a total of 140 VLBWIs were simultaneously analyzed with direct and indirect ISE methods. Among them, 21 VLBWIs with the use of diuretics and/or steroid, 14 with a hemolytic sample, and 9 with congenital anomalies people were excluded from this study. Finally, 106 VLBWIs were included in this study. Of them, 76 (71.7%) had sodium ion level difference between indirect and direct ISE methods exceeding 4 mmol/L at birth. Mean GA and birth weight were 29.4 ± 3.1 weeks and 1138.9 ± 270.6 g, respectively. Sodium ion level at birth measured with the indirect ISE method using a chemistry auto-analyzer was 138.6 ± 4.1 mmol/L. It was 132.6 ± 3.7 mmol/L when the direct ISE method with arterial blood gas analysis was used for measurement. A Bland–Altman comparison of sodium ion level between the 2 methods showed that limits of agreement ranged from −0.2 to 12.0 mmol/L (Fig. 1). Except for 1 patient, all included VLBWIs showed higher sodium ion levels at birth by indirect the ISE method than by the direct ISE method. The mean difference of sodium ion level (indirect – direct ISE sodium ion level) was 5.9 ± 6.1 mmol/L, which was significantly (*P* < .05) higher than 4 mmol/L.

Patients were divided into 2 groups based on threshold of 4 mmol/L for sodium ion level difference. GA was 29.0 ± 2.8 weeks for the group with sodium ion level difference >4 mmol/L group. It was 30.5 ± 3.8 weeks for the group with sodium ion level difference ≤4 mmol/L group, showing a significant (*P* < .05) difference between the 2 groups. Although SGA was high for the sodium ion level difference ≤4 mmol/L group, it was not significantly different between the 2 groups. Except for these factors, other characteristics and maternal factors were not significantly different between 2 groups (Table 1).

**Table 1:** Demographic characteristics of VLBWIs.

Characteristics	Total (N = 106)	Sodium gap >4 (N = 76)	Sodium gap ≤4 (N = 30)	*P*-value
Gestational age, wk	29.4 ± 3.1	29.0 ± 2.8	30.5 ± 3.8	<.05
Birth weight, g	1138.9 ± 270.6	1118.3 ± 255.5	1190.9 ± 303.9	.22
Small for gestational age, n (%)	20 (18.9)	11 (14.5)	9 (30.0)	.07
Male, n (%)	48 (45.3)	35 (46.1)	13 (43.3)	.83
Caesarean section, n (%)	73 (68.9)	53 (69.7)	20 (66.7)	.82
Maternal age, year	33.1 ± 5.1	33.2 ± 5.0	32.9 ± 5.2	.80
Multiple gestation, n (%)	22 (20.8)	14 (18.4)	8 (26.7)	.35
Completed antenatal steroid, n (%)	65 (61.3)	48 (63.2)	17 (56.7)	.54
In vitro fertilization, n (%)	26 (24.5)	18 (23.7)	8 (26.7)	.75
Maternal antibiotics, n (%)	13 (12.3)	11 (14.5)	2 (6.7)	.34
Clinical chorioamnionitis, n (%)	20 (18.9)	15 (19.7)	5 (16.7)	.72
Gestational diabetes mellitus, n (%)	7 (6.6)	6 (7.9)	1 (3.3)	.67
Pregnancy associated hypertension, n (%)	57 (53.8)	42 (55.2)	15 (50.0)	.90
Preterm premature rupture of membranes, days	3.4 ± 1.0	2.6 ± 8.8	5.4 ± 12.8	.19

Table 2 shows the clinical factors and blood test at birth between the 2 groups. Among neonatal clinical factors, the 1-minute Apgar score was lower (*P* < .05) in the sodium ion level difference >4 mmol/L group. There was no difference in vital sign or mortality-predicting score such as clinical risk index of babies II, SNAP II, or SNAP perinatal extension score between the 2 groups. The sodium ion level difference >4 mmol/L group showed higher chemistry sodium level by the indirect ISE method, lower albumin level, and lower protein level than the other group (Table 2).

**Table 2:** Clinical features and laboratory data of VLBWIs at birth.

Variables	Sodium gap >4 (N = 76)	Sodium gap ≤4 (N = 30)	*P*-value
1-min Apgar score	4.76 ± 2.05	5.60 ± 1.73	<.05
5-min Apgar score	6.79 ± 1.95	7.33 ± 1.24	.09
Body temperature at admission, °C	35.79 ± 0.85	35.84 ± 0.65	.80
FiO2 at admission, %	31.54 ± 20.38	28.33 ± 15.86	.44
Urine output during first 24 h, mL/kg/h	1.76 ± 1.27	2.20 ± 1.22	.11
CRIB II score	8.57 ± 3.40	7.60 ± 3.43	.19
SNAP II score	20.00 ± 13.46	15.37 ± 11.70	.10
SNAPPE score	32.38 ± 21.87	24.97 ± 19.46	.11
Chemistry sodium, mmol/L	139.67 ± 3.86	135.70 ± 3.49	<.05
ABGA sodium, mmol/L	132.30 ± 3.84	133.49 ± 3.32	.14
Protein, g/L	41.43 ± 6.38	47.40 ± 6.29	<.05
Albumin, g/L	27.61 ± 4.09	30.40 ± 3.77	<.05

Neonatal clinical features within the first week of life were compared between the 2 groups in Table 3. Input volume and amount of urine output within the first week of life did not show any significant difference between the 2 groups. Duration of phototherapy within a week was longer in the sodium ion level difference >4 mmol/L group (4.2 ± 1.6 vs 3.1 ± 1.9 days, *P* < .05). The sodium ion level difference >4 mmol/L group showed a higher maximum percent of physiological weight loss than the other group (*P* < .05). Except for this, the 2 groups showed no significant difference in neonatal disease (IVH, hemodynamic significant PDA, RDS, or early sepsis). Sodium, potassium, and chloride ion levels on the seventh day did not show a significant difference between the 2 groups either.

**Table 3:** Comparison of clinical features within the first week of life and laboratory data.

Variables	Sodium gap >4 (N = 76)	Sodium gap ≤4 (N = 30)	*P*-value
Intraventricular hemorrhage (grade ≥3), n (%)	6 (7.9)	1 (3.3)	.67
HS PDA, n (%)	24 (31.6)	10 (33.3)	1.00
Respiratory distress syndrome, n (%)	61 (80.3)	20 (66.7)	.11
Early sepsis, n (%)	1 (1.3)	1 (3.3)	.49
Percent of maximum weight loss, %	10.6 ± 5.0	7.4 ± 5.2	<.05
Days of ventilator care until 7 d of life, day	3.4 ± 3.0	2.7 ± 2.9	.31
First week input volume, mL	83.6 ± 11.3	86.1 ± 11.6	.10
First week urine output, mL/kg/d	2.8 ± 0.6	2.7 ± 0.3	.31
Death within first week of life, n (%)	7 (13.2)	0 (0.0)	.19
Plasma sodium, mmol/L	136.4 ± 5.2	135.5 ± 3.3	.41
Plasma potassium, mmol/L	4.9 ± 1.0	4.6 ± 0.8	.28
Plasma chloride, mmol/L	112.0 ± 4.8	111.6 ± 4.1	.31
Whole blood sodium, mmol/L	142.2 ± 6.7	140.1 ± 4.1	.12
Whole blood potassium, mmol/L	5.2 ± 0.9	4.9 ± 0.6	.10
Whole blood chloride, mmol/L	112.8 ± 6.8	111.8 ± 3.2	.32

Logistic regression analysis was performed for clinical factors associated with sodium ion level difference >4 mmol/L between direct and indirect ISE methods. After adjusting for GA and SGA that showed significant difference between the 2 groups, protein level at birth (odds ratio: 0.835, 95% confidence interval: 0.760–0.918, *P* < .001) and percent of maximum weight loss (odds ratio: 1.137, 95% confidence interval: 1.021–1.265, *P* = .019) were found to be factors associated with sodium ion level difference >4 mmol/L between direct and indirect ISE methods (Table 4).

**Table 4:** Odds ratios for the risk of sodium ion concentration exceeding 4 mmol/L with respect to clinical factors.

	Unadjusted			Adjusted		
Variables	OR	CI 95%	*P*-value	OR	CI 95%	*P*-value
Gestational age, week	0.860	0.748–0.988	.034	1.127	0.900–1.411	.298
Small for gestational age	1.898	0.718–5.020	.197	0.668	0.179–2.498	.549
Albumin, g/L	0.848	0.757–0.950	.004	0.917	0.723–1.164	.476
Protein, g/L	0.858	0.792–0.930	<.001	0.835	0.760–0.918	<.001
1-min Apgar score	0.826	0.660–1.033	.093	0.893	0.633–1.259	.518
5-min Apgar score	0.870	0.675–1.123	.285	1.330	0.725–2.440	.357
Percent of maximum weight loss, %	1.131	1.034–1.238	.007	1.137	1.021–1.265	.019

## 4 Discussion

In the present study, the maximum percentage of physiology weight loss and plasma protein level were found to be factors associated with sodium ion level difference >4 mmol/L between direct and indirect ISE methods on the first day after birth. Previous studies have also shown that protein in blood is correlated with sodium ion difference between the 2 ISE methods.^[4,9,12,13]^ However, correlations between sodium ion level difference between the 2 ISE methods and clinical factors in preterm infants have not been reported yet.

The amount of weight loss in the first week of the life of VLBWIs was found to be similar to the previous studies, showing that 7.9% to 14.6% of birth weights were lost during the first week of life.^[14,15]^ The prevalence rate of neonatal diseases such as RDS and hemodynamically significant PDA occurring within the first week of life in preterm infants could be increased by hypervolemia.^[16–18]^ These diseases and electrolytes at seventh days were not significantly different between the 2 groups. There was no significant difference in input or urine output volume within a week. However, there was a difference in the duration of phototherapy. Although this study was designed retrospectively, fluid management was consistently controlled.

Preterm infants experience a decrease in extracellular fluid (ECF) volume through natriuresis and diuresis after birth.^[19]^ As a result, they undergo physiologic weight loss with ECF contraction of body fluid.^[20]^ Considering that the duration of phototherapy was longer for the group with sodium ion level difference between direct and indirect ISE methods >4 mmol/L and do not differ in input or urine output volume, it could be inferred that physiologic weight difference between the 2 groups was caused by IWL. Meanwhile, during the postnatal adaptation period, weight loss tends to attenuate for SGA patients.^[21]^ The reason why the group with sodium ion level difference >4 mmol/L had more SGA patients might be association between increased physiologic maximum weight loss and sodium ion level difference.

In addition to the previously known associated factors such as plasma protein, this study revealed that sodium difference between direct and indirect ISE methods was independently associated with initial maximum weight loss. Since we simultaneously analyzed collected arterial blood samples, sodium difference between 2 ISE methods was not related to difference in a discrepancy between capillary and/or arterial and/or venous blood. Sodium difference was likely to be associated with solid components in the blood such as lipid and other unknown factors. The result of this study showed that the difference in sodium ion level between the 2 groups was mainly due to the chemistry sodium ion level measured with the indirect ISE method. Solid component also makes incorrect sodium ion in indirect ISE method. Unmeasured solid component might make the sodium level difference between direct and indirect ISE methods. These components might also be associated with physiologic weight loss in VLBWIs. Namely, the association could be presented as a way to predict physiologic weight changes.

To date, there is no way to predict the amount of physiology weight loss at right after birth. In clinical practice, fluid management is based on subjective indicators including the condition of a patient visible to naked eyes and the neonatologist's experience rather than based on objective data. As a result, the prediction of maximum weight loss at birth could reduce unnecessary fluid intake that might increase mortality and morbidities such as PDA and bronchopulmonary dysplasia. If VLBWIs’ maximum physiology weight loss meaning ECF volume of body water is predictable with sodium ion at birth, it will help fluid management in the first week of life. If there is a substance associated with sodium ion level difference between direct and indirect ISE methods, it is necessary to further study about the association with physiologic weight loss.

This retrospective study has some limitations. According to our strict NICU policy, we tried to keep volume appropriate management considering IWL and urine amount. Although, input volume and urine output were not significantly different between the 2 groups, we could not calculate IWL during first week of life. IWL difficult to measure, such as phototherapy, maybe make physiologic weight loss between 2 groups. And antidiuretic hormone and renin-angiotensin-aldosterone system associated hormone those are released with urine amount were not measured. These unmeasured hormonal changes could be an important factor that made sodium level difference. Finally, although this study reflected the condition up to 1 week after birth, it had a limit to reflect overall sodium difference.

In conclusion, difference in sodium ion level between direct and indirect ISE methods in the early stages of life might reflect the extent of maximum physiology weight loss in VLBWIs. Based on this study, if the study to predict the body's composition of ECF and intracellular fluid is proceeded, it will help neonatologist make clinical decisions at early life of preterm infants.

## Author contributions

**Conceptualization:** Jin Kyu Kim.

**Data curation:** Jin Kyu Kim.

**Formal analysis:** Hyun Ho Kim.

**Funding acquisition:** Jin Kyu Kim.

**Methodology:** Hyun Ho Kim.

**Writing – original draft:** Hyun Ho Kim.

**Writing – review & editing:** Jin Kyu Kim.
